# A New Method for the Treatment of Ingrowing Toe-Nails

**Published:** 1885-02-20

**Authors:** T. D. King

**Affiliations:** Student of Medicine, University of Pennsylvania


### A NEW METHOD FOR THE TREATMENT OF INGROWING TOE-NAILS.

By T. D. King, A. M.,
Student of Medicine, University of Pennsylvania.

Probably it would be more correct to speak of an overgrowing fold of skin, rather than an ingrowing toe-nail, for the former expression more accurately describes the condition of things in most, if not all, such cases.

This overgrowing of the skin is caused, usually, by the pressure of the adjacent toe ; or, if on the outside or the inside of the foot, by the pressure of an ill-fitting shoe. It was the observance of this fact, followed by attempts to counteract or remove this pressure, that finally led to the mode of treatment here suggested.

Scrape or gouge out a narrow groove in the center of the nail from its base to its free margin, making the nail so thin that when either side is lifted it will turn on this groove as on a hinge, and will not press the opposite side of the nail down into the flesh. Force a small piece of cotton under the edge of the nail on the sound side of the toe. Then take an oblong piece of cotton, as long as you judge you can possibly use for the purpose, and force the *centre* of it as far under the irritating point and buried edge of the ingrowing nail as the patient can bear to have it. A very gradual, interrupted introduction of the cotton will cause the minimum of pain to the patient. Then pack one end of the cotton around under the lower margin of the nail. Fold the other end back upon the nail, and push it as much as possible under the flesh at the side and base of the ingrowing nail.

Up to this point there is nothing new in treatment, except, perhaps, the folding over of one end of the cotton so as to form a compress along the inner side of the flesh which has overlapped the ingrowing nail.

Now, take a bandage of muslin, as broad as the toe is long, and about a foot and a half in length, and place the initial extremity underneath the affected toe. Carry it up on the sound side of the toe, over the toe, down over the ingrowing nail, and beneath the toe which lies against the ingrowing nail; then up on the far side, and back over the top of that toe; over the top of the affected toe again, down on the sound side, and under it, thus fixing the initial extremity of the bandage.

Then, lifting the adjacent toe in the loop you have made around it, pull it as far as possible up over the top of the affected toe, and fasten it there by carrying the bandage beneath both toes and making circular turns around the two, held in that position until the bandage is exhausted. Having made as firm pressure as possible without causing pain, fasten the bandage with a pin on top and between the two toes.

Often the bandage may have to be comparatively loose when first applied, but in a day or two it will be found that it can be made very tight without discomfort or pain.

When first put on, this bandage will feel very uncomfortable to the patient; but, if a well-fitting shoe (not a tight shoe) is worn, and the patient goes about his or her usual occupation, in an hour or two this uncomfortable feeling wears off, and the ingrowing nail is forgotten.

Fresh cotton should be put under the nail every day, in the manner described, till it can be pushed back under the edge of the ingrowing nail almost to its base. After that the cotton need be changed but once a week, unless it becomes wet from any cause, when it should be immediately changed.

The bandage may be removed at bedtime, and replaced on arising in the morning, if it occasions any discomfort when worn at night.

The groove in the center of the nail must be constantly renewed as the nail grows out; and the nail must be trimmed square across when it gets too long for comfort. It should never be trimmed closely, nor should the corners ever be rounded off in trimming it.

This treatment ought to be continued until the adjacent toe has acquired the habit of remaining above the ingrowing nail instead of below it, for it will be found that the majority of ingrowing nails are caused by the adjacent toe, which presses the flesh up over the ingrowing nail.

As will be readily perceived, the effect of this treatment is, in the first place, to press the flesh down and away from the ingrowing nail by means of the superimposed toe; and, secondly, by means of the upward pressure of the cotton under the edges of the nail on either side, together with the hinge-like action of the central groove, to encourage the nail to spread out over the top of the toe.

The flesh over the ingrowing nail is pressed down, and forms a teat like projection at the side of the toe, which rapidly atrophies and disappears, leaving the ingrowing nail bared, and free to spread out. The compress, formed of one end of the piece of cotton used, aids in this very much.

The removal of the pressure of the adjacent toe (which is usually the chief cause of the ingrowing toe-nail, and which often prevents a permanent cure), together with the use of that toe in exerting pressure in the opposite direction, are the essentially new points in this treatment.

The results, wherever this treatment is faithfully carried out, are most satisfactory. Immediate and entire relief is given to the patient, and the case goes on to a permanent cure, requiring little care and causing no pain or discomfort after the first few days. The writer has seen these results repeatedly in patients whose business has kept them on their feet for a large part of the time during the treatment.

Doubtless cases of ingrowing nail will not yield to this treatment; yet we believe that many cases can be cured more rapidly and more pleasantly by this method than by any other conservative treatment now in vogue.

It will be observed that this method of treatment cannot be used when the ingrowing nail is on the inner side of the big toe, or on the outer side of the little toe. In such cases, the nearest approach possible to this treatment will be the use of a piece of cork in the manner suggested by Professor Agnew.—*Med. and Surg. Rep.*

[Where these methods fail, and an operation becomes necessary, the operation recommended by Dr. R. C. Word, in the SOUTHERN MEDICAL RECORD for January, 1884, is most simple and satisfactory.—ED. RECORD.]

